# The Proline Dehydrogenase Gene *CsProDH1* Regulates Homeostasis of the Pro-P5C Cycle Under Drought Stress in Tea Plants

**DOI:** 10.3390/ijms26073121

**Published:** 2025-03-28

**Authors:** Deng Deng, Qinqin Gao, Rou Zeng, Jie Jiang, Qiang Shen, Yuanchun Ma, Wanping Fang, Xujun Zhu

**Affiliations:** 1 College of Horticulture, Nanjing Agricultural University, Nanjing 210095, China; 2022104085@stu.njau.edu.cn (D.D.); 2022104086@stu.njau.edu.cn (Q.G.); 2023104079@stu.njau.edu.cn (R.Z.); jiangjie@njau.edu.cn (J.J.); myc@njau.edu.cn (Y.M.); fangwp@njau.edu.cn (W.F.); 2 Tea Research Institute, Guizhou Provincial Academy of Agricultural Sciences, Guiyang 417100, China; shenqiang_gzu@163.com

**Keywords:** proline dehydrogenase, Pro-P5C cycle, drought stress

## Abstract

The homeostasis of the proline-Δ^1^-pyrroline-5-carboxylate (Pro-P5C) cycle, mediated by proline dehydrogenase (ProDH), plays a critical role in plants in response to abiotic stresses. The biological function of gene *CsProDH1* under drought stress and its effects on amino acid metabolism and photosynthesis through proline metabolism were investigated. Enzymatic characterization of the CsProDH1 protein was conducted in vitro. Overexpression of *CsProDH1* aggravated plant stress, as evident by reduced photosynthetic efficiency and increased reactive oxygen species, which activated the Pro-P5C cycle. In contrast, silencing *CsProDH1* enhanced plant drought resistance, increased proline accumulation, and protected photosynthesis. Studies indicate that exogenous amino acid application mitigates drought-induced physiological impairments in plants by maintaining cellular homeostasis, with particular efficacy observed in enhancing tea plant drought resilience through improved osmotic adjustment and antioxidant capacity. This study uncovers the significant role of *CsProDH1* in plant drought resistance and its regulatory mechanism, offering potential gene targets and application strategies for enhancing crop drought resistance.

## 1. Introduction

Drought is an important factor limiting agricultural production and severely affecting crop yields [1]. Plants mitigate drought stress by activating drought response mechanisms, such as morphological and structural changes, expression of drought-resistant genes, and synthesis of hormones and osmotic regulatory substances [2,3]. Many studies have found that plants accumulate proline to stabilize subcellular structures and maintain internal balance [4]. Proline is generally considered a unique low-molecular-weight osmolyte that can cope with various osmotic-related stresses in plants, such as drought stress and salt stress [5,6].

The concentration of free proline in plant cells is primarily determined by four metabolic processes: the biosynthesis and degradation of proline, as well as the consumption of proline in protein biosynthesis and its release during protein degradation [7]. Proline in plants is synthesized through two pathways: the glutamate (Glu) and ornithine [8]. The Glu pathway is presumed to be the main synthetic metabolic branch under osmotic stress. In the Glu pathway, the bifunctional enzyme Δ^1^-pyrroline-5-carboxylate synthase (P5CS) converts L-Glu into glutamate-γ-semialdehyde (GSA), which spontaneously cyclizes to form Δ^1^-pyrroline-5-carboxylate (P5C), which is then catalyzed by Δ^1^-pyrroline-5-carboxylate reductase (P5CR) to generate proline [8,9]. The ornithine pathway serves as an alternative route for proline formation, utilizing the initial steps of the arginine biosynthetic pathway to generate ornithine, which is then converted to P5C by ornithine δ-aminotransferase (OAT) and finally reduced to proline by ornithine cyclodeaminase or directly converted to proline. In some organisms, this latter pathway contributes to or may entirely be responsible for proline synthesis.

The intermediate products of proline metabolism, P5C/GSA, can be shared by both catabolic and anabolic pathways, but the degradation of proline is not entirely a reverse reaction of its synthesis, and the enzymes catalyzing the reactions also differ [10,11]. Catabolism of proline mainly occurs in mitochondria through the action of proline dehydrogenase (ProDH) and Δ^1^-pyrroline-5-carboxylate dehydrogenase (P5CDH) [12]. Proline is oxidized by ProDH to produce P5C, which is then converted to Glu by P5CDH, providing fuel for mitochondrial respiration and the tricarboxylic acid (TCA) cycle [13]. Zhao et al. showed that *ProDH* may be one of the key genes affecting drought tolerance in plants through transcriptomics and metabolomics data [14]. During abiotic stress, proline degradation is inhibited because *ProDH* transcription is suppressed by dehydration, but after the plant is relieved from stress, the degradation process of proline returns to normal [15,16]. In the mitochondria, proline is first oxidized to P5C by ProDH, and then P5C is converted to Glu under the action of P5CDH [17]. The ProDH and P5CDH are key in the degradation process of plant proline. As proline degradation proceeds, proline accumulation in the plant body decreases, gradually returning to normal levels, and the osmotic balance within plant cells tends to stabilize [13].

Enzymes ProDH and P5CDH, which are involved in proline oxidation, are highly conserved in both eukaryotic and prokaryotic organisms. Studies have found that proline degradative enzymes can be divided into three categories: the first category consists of monofunctional enzymes, where the ProDH domain and P5CDH domain are separate; the second and third categories both contain ProDH and P5CDH domains within the same PutA polypeptide, with the distinction being the presence or absence of a ribbon–helix–helix (RHH) DNA-binding domain at the N-terminus. The PutA contains the DNA-binding domain and has three functions. In addition to possessing ProDH and P5CDH enzymatic activities, the RHH domain enables PutA to act as a transcriptional regulator for the PutA and PutP (encoding a high-affinity Na^+^/proline transporter) genes [18]. It was previously believed that proline degradation in eukaryotes relied on monofunctional enzymes ProDH and P5CDH, while in prokaryotes, it depended on bifunctional PutA. Current research indicates that proline degradative enzymes in Gram-positive bacteria are monofunctional, whereas those in Gram-negative bacteria are bifunctional PutA [19]. In *Arabidopsis thaliana*, two *ProDH* genes have been identified and functionally characterized. The *AtProDH1* is a dehydration-responsive gene that is upregulated upon rehydration, coinciding with a decrease in intracellular proline [20]. *AtProDH1* appears to be the predominant isoform, while *AtProDH2* is specifically upregulated during salt stress [21].

Both ProDH and P5CDH are mitochondrial enzymes that utilize flavin adenine dinucleotide (FAD) and nicotinamide adenine dinucleotide (NAD^+^) as electron acceptors, producing, respectively, FADH_2_ and nicotinamide adenine dinucleotide phosphate (NADH) to provide electrons for mitochondrial respiration [22]. ProDH has been detected in the cytoplasm of soybean root nodules, indicating that proline oxidation provides energy for rhizobia during nitrogen fixation [23]. Proteomic analysis has identified P5CDH in chloroplasts, suggesting that P5C can also be converted to Glu in the plastids [24]. Recent studies have shown that P5C produced from proline in the mitochondria can be transported to the cytoplasm and reduced back to proline by cytoplasmic P5CR. When P5CDH activity is limited, the Pro-P5C cycle can transfer more electrons to the mitochondrial electron transport chain, generating reactive oxygen species (ROS). Overactivation of this cycle under conditions of excess exogenous L-proline leads to ROS production through electron transfer to oxygen (O_2_). Inhibition of P5CDH activity results in increased ROS production due to proline accumulation. Therefore, balancing ROS production during oxidation is crucial for avoiding proline-related toxic effects [25].

Pro biosynthesis is upregulated by light and osmotic stress, while its catabolism is activated during darkness and stress relief, controlled by ProDH and P5CDH. ProDH is activated by rehydration and Pro is inhibited by dehydration, preventing Pro degradation during abiotic stress. *ProDH* is suppressed during the day and induced in darkness, with contrasting effects of light on *P5CS* and *ProDH* transcription [26]. After stress relief, ProDH and P5CDH oxidize proline to provide reducing equivalents to the mitochondria, supplying electrons to the respiratory chain and aiding in the recovery of energy for growth. The recently discovered Pro-P5C cycle can transfer electrons to the mitochondrial electron transport chain without producing Glu and, under certain conditions, can generate more ROS in the mitochondria [25,27]. When P5C oxidation is impaired, P5C can be exported to the cytosol via an unknown transporter and reduced back to proline by P5CR, forming the Pro-P5C cycle [28]. The production of ROS by the Pro-P5C cycle was initially confirmed in animal cells [29,30], where ProDH leads to mitochondrial ROS accumulation and apoptosis in a proline-dependent manner [31,32]. In plants, it has been proposed that P5C is exported from the mitochondria, and the activation of the Pro-P5C cycle has been described in *ProDH*-overexpressing or *P5CDH A. thaliana* mutant plants, where increased electron flow to the mitochondrial electron transport chain stimulates the cycle, resulting in increased mitochondrial ROS [25,33]. The cycle operates when the increase in ProDH activity is not matched by an increase in P5CDH activity, leading to incomplete oxidation of Pro and accumulation of P5C in the mitochondria. In this scenario, P5C is exported to the cytosol, converted back to proline by P5CR, and then Pro is reincorporated into the mitochondria to restart the cycle [34].

Tea (*Camellia sinensis* L.) is one of the most popular non-alcoholic beverages, containing a variety of polyphenolic compounds that are beneficial to human health, making it a very important economic crop [35]. However, drought stress severely affects the yield and quality of tea leaves. In this study, we identified the *CsProDH* gene in the tea plant genome and investigated the effects of *CsProDH1* overexpression or silencing on plants under drought stress. Our data indicate that when *CsProDH1* is overexpressed, the activity of the Pro-P5C cycle is enhanced, leading to increased ROS.

## 2. Results

### 2.1. Expression Pattern of CsProDH1 Under Different Treatments

**Figure 1:**
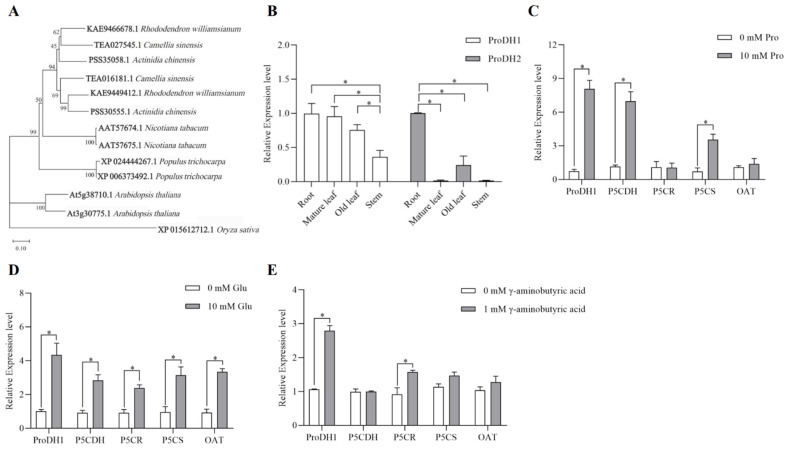
Expression patterns of tea plant *CsProDH1* under different treatments. (**A**) Phylogenetic evolutionary tree of ProDH in different species; (**B**) expression of tea plant *ProDH* gene in different tissues; (**C**) expression of Pro metabolism-related genes in tea plant mature leaves after exogenous Pro treatment; (**D**) expression of Pro metabolism-related genes in tea plant mature leaves after exogenous Glu treatment; and (**E**) expression of proline metabolism-related genes in tea plant mature leaves after exogenous γ-aminobutyric acid treatment. The data are the mean ± SD (*n* = 3). Significance was verified by *t*-tests; ‘*’ represents significance at *p* ≤ 0.05.

Based on the comparative screening of the tea plant genome database, the tea plant *ProDH* gene sequences TEA027545.1 and TEA016181.1 were obtained and named *CsProDH1* and *CsProDH2*, respectively. Phylogenetic trees were constructed using neighbor-joining in MEGA 6.0 software, and evolutionary relationships of tea plant CsProDH protein family were compared with *A. thaliana*, tobacco (*Nicotiana tabacum* L.), rice (*Oryza sativa* L.), round-leaved azalea (*Rhododendron williamsianum* Rehd. et Wils.), Chinese kiwi (*Actinidia chinensis* Planch.), and *Populus trichocarpa* (Figure 1A). The amino acid sequences encoded by the *CsProDH* family are closely related to those of *Rhododendron williamsianum* and *Actinidia chinensis*.

Expression of proline dehydrogenase genes in different tissues of tea plants differed in the following way: *CsProDH1* was expressed in roots, stems, and leaves, with higher expression in roots and leaves, while *CsProDH2* had its highest expression in roots (Figure 1B). Since the economic value of the tea plant is dominated by leaves, *CsProDH1* was used in the subsequent study. After applying exogenous amino acids, including Pro, Glu, and γ-aminobutyric acid, the expression levels of Pro metabolism genes in tea plants were investigated. The concentrations of different exogenous amino acids were determined through pre-experiments (Supplementary Figures S1–S3). After applying exogenous Pro, expression levels of *CsProDH1*, *CsP5CDH*, and *CsP5CS* increased (Figure 1C). The application of exogenous Glu resulted in significant increases in expression levels of all genes related to the Pro metabolic pathway (Figure 1D). Application of exogenous γ-aminobutyric acid resulted in increased expression of *CsProDH1* and *CsP5CR*, and expression levels of *CsP5CDH* and *CsP5CS* showed no significant change (Figure 1E).

### 2.2. Enzymatic Characterization of the CsProDH1 Protein

**Figure 2:**
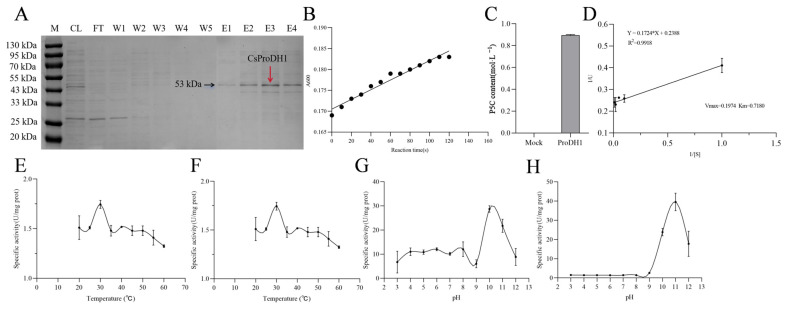
Enzymatic characterization of tea plant CsProDH1 protein. (**A**) The purification effect of His-tagged recombinant protein under denaturing conditions. M, marker; CL, bacterial lysate (cell lysate); FT, flow-through; W1–W3, denaturing lysate washing 1–3; W4 and W5, denaturing washing solution washing; E1–E4, eluent 1–4; (**B**) results of the enzymatic characterization of CsProDH1 protein; (**C**) results of in vitro protein function validation of CsProDH1; (**D**) Lineweaver–Burke double inverse plots of CsProDH1 enzyme kinetics; (**E**) enzyme activity of CsProDH1 at different temperatures; (**F**) temperature stability of CsProDH1; (**G**) CsProDH1 enzyme activities at different pH; and (**H**) pH stability of CsProDH1.

The *CsProDH1* was constructed on the prokaryotic expression vector pet-32a and then transformed into *Escherichia coli* BL21 sensory state, and isopropyl-beta-d-thiogalactopyranoside (IPTG) was added to the Luria–Bertani (LB) liquid medium to induce expression of the CsProDH1-His tag fusion protein. A small amount of the obtained bacterial lysate, supersample flow-through, denaturing lysate wash, denaturing wash, and eluent from the purification process of CsProDH1-His-tagged protein were used for Sodium Dodecyl Sulfate–Polyacrylamide Gel Electrophoresis (SDS-PAGE) (Figure 2A). The eluate obtained was collected as the purified His-tagged protein sample.

The enzyme activity assay used a published method [36], and the change in absorbance value at 600 nm per unit time was determined using L-Pro as the substrate. When the enzyme activity assay reaction started, the absorbance value of the reaction solution at 600 nm increased continuously with reaction time, while the absorbance of the control solution showed no change in the corresponding time (Figure 2B). The P5C content of the reaction solution was determined using an enzyme marker. After sufficient reaction, no P5C was detected in the control, and P5C was produced in the CsProDH1 (Figure 2C). This indicates that the purified CsProDH1 protein had ProDH activity.

A linear equation fitted to a Lineweaver–Burk plot showed that CsProDH1 had a maximum velocity (Vmax) value of 0.1974 μmol·mg^−1^ and a Michaelis constant (Km) value of 0.7180 mM using proline as the substrate (Figure 2D). The enzyme activity assay (Figure 2E) showed that CsProDH1 activity increased dramatically with the increase in temperature within 20–30 °C, reached a peak at 30 °C, then declined at 35 °C, but again increased at 40 °C, after which it decreased rapidly with increased temperature. With rising potential of hydrogen (pH) values, CsProDH1 activity gradually increased within pH 3–8, reaching a peak at pH 10.0, after which it rapidly decreased (Figure 2G). Temperature stability analysis showed that, with increased temperature, CsProDH1 maintained maximum enzyme activity under incubation at 30 °C, after which the activity declined with further temperature elevation. (Figure 2E). The overall trend in enzyme activity after incubation at 20–30 °C was elevated. The pH stability analysis showed that CsProDH1 had the highest activity at pH 11.0 (Figure 2H) but was close to inactivation after incubation at pH 3.0–9.0.

**Figure 3:**
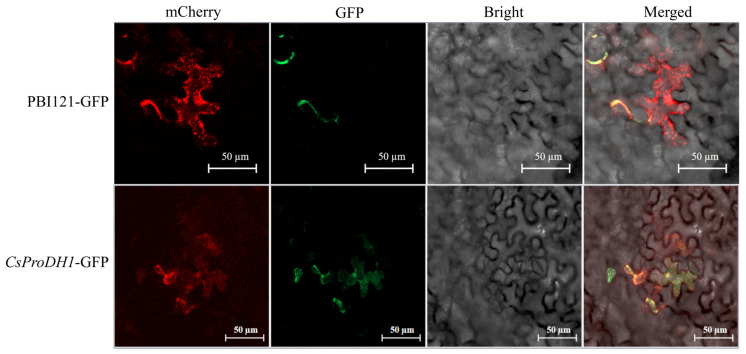
Intracellular localization of the CsProDH1 protein. Scale bar = 50 μm.

The location of CsProDH1 was predicted to be mitochondrial through multiple sites. To confirm the prediction results, we constructed a vector with a fusion of the target gene fragment and green fluorescent protein (GFP)-tagged protein and, using the *Agrobacterium*-mediated method, we mixed the recombinant plasmid with a mitochondrial marker for tobacco subepidermal injection using the empty vector pBI121-GFP as a control and observed it under a laser confocal microscope. The control empty vector pBI121-GFP protein was not present in mitochondria, while the fluorescent signal of *CsProDH1*-GFP was present in mitochondria (Figure 3), consistent with the predicted results.

### 2.3. Transient Expression Assay of CsProDH1

**Figure 4:**
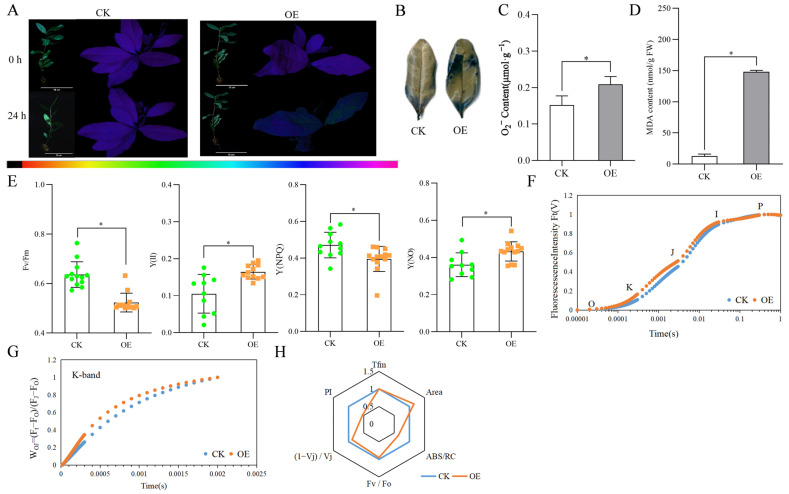
Changes in parameters related to overexpression treatment of *CsProDH1* in tea plants under drought conditions. (**A**) Phenotype and in vivo fluorescence imaging at 24 h processing. Scale bar = 10 cm; (**B**) leaf NBT staining at 24 h processing; (**C**) leaf O_2_^−^ content at 24 h processing; (**D**) leaf MDA content at 24 h processing; (**E**) parameters related to in vivo fluorescence imaging at 24 h processing. Fv/Fm, maximum photosynthetic efficiency; Y(II), actual photosynthetic efficiency of PSII; Y(NPQ), quantum yield of regulated energy dissipation in PSII; and Y(NO), quantum yield of non-regulated energy dissipation in PSII. CK, green circle; OE, orange square. (**F**) OJIP curves; (**G**) fluorescence rise kinetics normalized by F_0_ and F to W_OJ_ = (F_t_ − F_0_)/(F − F_0_); and (**H**) radar plot of fluorescence parameters. Tfm, the time required from dark adaptation to illumination to maximum fluorescence; Area, the OJIP curve, fluorescence intensity F = F_m_, and the area between the Y-axis; F_v_/F_0_, the ability of the PSII reaction center to capture and convert light energy; ABS/RC, light energy absorbed per reaction center; (1 − Vj)/Vj, the efficiency/probability with which an electron from the intersystem electron carriers is transferred to reduce end electron acceptors at the PSI acceptor side; PI, performance index. CK, control group; OE, overexpression group. The data are the mean ± SD (*n* = 3). Significance was verified by *t*-tests; ‘*’ represents significance at *p* ≤ 0.05.

The composite treatment of drought stress + overexpression of *CsProDH1* resulted in the leaves of the overexpression group showing green fluorescence compared with the control group, indicating that the overexpression group was subjected to a deeper degree of stress (Figure 4A). Tea plant leaves were stained using the nitro blue tetrazolium (NBT) staining method (Figure 4B). Compared with drought-stressed tea plants, *CsProDH1*-overexpressing tea plant leaves had more blue spots in NBT staining and also significant increases in contents of superoxide (O_2_^−^) and malondialdehyde (MDA) (Figure 4C,D). Transient overexpression effect of the *CsProDH1* gene is shown in Supplementary Materials (Figure S4).

The photosystem Ⅱ complex (PSII) maximum quantum efficiency [Fv/Fm], PSII effective photochemical quantum yield [Y(II)], PSII non-regulated energy dissipation [Y(NO)], and PSII regulated energy dissipation [Y(NPQ)] of the leaves of control and overexpression groups were analyzed. The results showed that Fv/Fm and Y(NPQ) were decreased, while Y(II) and Y(NO) were elevated in leaves of the overexpression group compared with the control (Figure 4E).

The effects of the composite treatment of drought stress and transient overexpression of *CsProDH1* on the fast chlorophyll a fluorescence induction (OJIP) curve of tea plant are shown in Figure 4F. The *CsProDH1* overexpression increased the K- and J-phase of the OJIP curve, and there was no significant difference between the I–P phase and the control group. Overexpression of *CsProDH1* increased the K-band (Figure 4G), suggesting that the exocytosis complex, oxygen-evolving complex, on the donor side of PSII in the overexpression group was more severely disrupted. The radar plot of fluorescence parameters showed that the overexpression group had lower PI and ABS/RC than the control group (Figure 4H).

### 2.4. CsProDH1 Gene Silencing Analysis Under Drought Treatment

**Figure 5:**
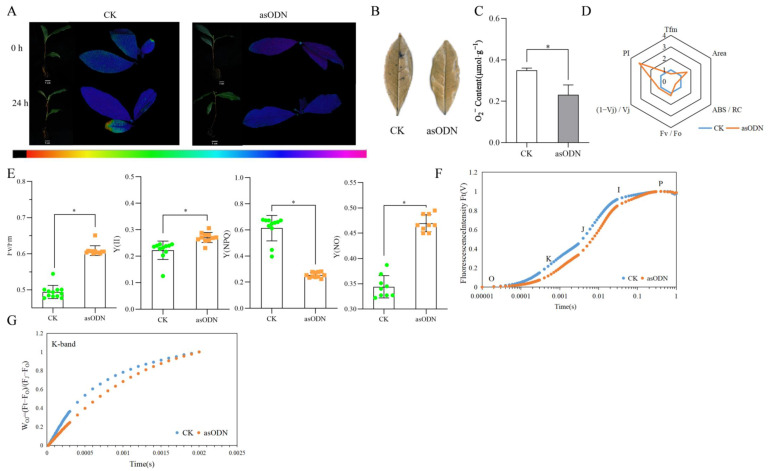
Changes in parameters related to transient silencing treatment of *CsProDH1* in tea plants under drought conditions. (**A**) Phenotype and in vivo fluorescence imaging. Scale bar = 10 cm; CK, green circle; asODN, orange square (**B**) leaf NBT staining at 24 h processing; (**C**) leaf O_2_^−^ content at 24 h processing; (**D**) radar plot of fluorescence parameters at 24 h processing; (**E**) parameters related to in vivo fluorescence imaging at 24 h processing; (**F**) OJIP curves at 24 h processing; and (**G**) fluorescence rise kinetics normalized by F_0_ and F to W_OJ_. CK, control group; asODN, silencing group. The data are the mean ± SD (*n* = 3). Significance was verified by *t*-tests; ‘*’ represents significance at *p* ≤ 0.05.

The composite treatment of drought stress + *CsProDH1* gene silencing resulted in less green fluorescence in the leaves of the silenced compared with the control group (Figure 5A), indicating that the silenced group was subjected to less stress. Tea plant leaves were stained using NBT staining (Figure 5B). Compared with drought-stressed tea plants, tea plant leaves after *CsProDH1* overexpression had more blue spots in NBT staining and also a significant decrease in O_2_^−^ content (Figure 5C). Instantaneous silencing effect of the *CsProDH1* gene in Supplementary Materials (Figure S5).

The Fv/Fm, Y(II), Y(NO), and Y(NPQ) of control and silenced group leaves were analyzed (Figure 5E). The Fv/Fm, Y(II), and Y(NO) were elevated while Y(NPQ) decreased in leaves of the silenced group compared with the control group. The OJIP curve for drought stress + *CsProDH1* gene silencing of tea plants showed that the K-, J-, and I-phases were reduced after *CsProDH1* silencing compared with the control group (Figure 5F). The K-band was reduced by *CsProDH1* silencing, indicating that the oxygen-evolving complex on the donor side of PSII in the silencing group was less damaged (Figure 5G). The radar plot of fluorescence parameters showed that PI, (1 − Vj)/Vj, and F_v_/F_0_ were greater in the silenced group than in the control, while ABS/RC was smaller than in the control (Figure 5D). This indicated that Pro accumulation in tea plant leaves was favorable for tea plants to resist drought stress.

### 2.5. Exogenous Application of Amino Acids for Recovery Treatment

**Figure 6:**
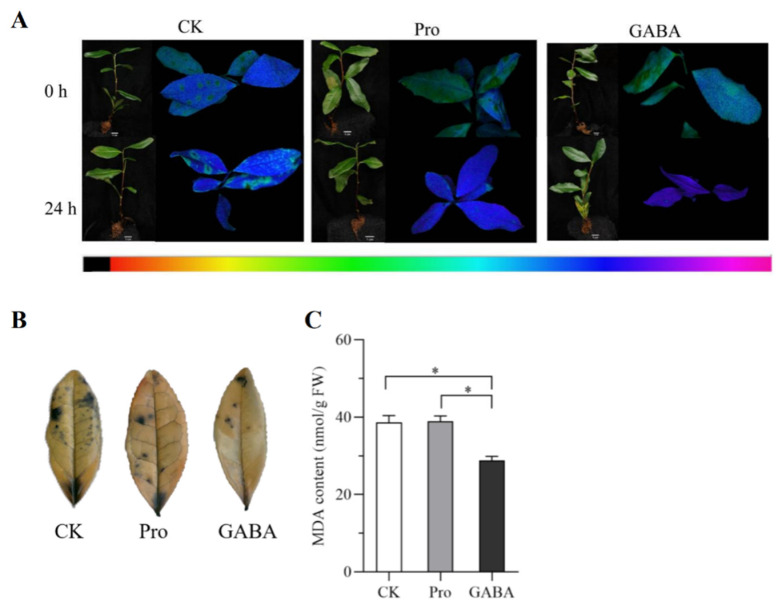
Exogenous application of amino acids for recovery treatment after 24 h of *CsProDH1* overexpression under drought conditions. (**A**) Phenotype and in vivo fluorescence imaging. Scale bar = 1 cm; (**B**) leaf NBT staining 24 h after spraying with externally supported amino acids; and (**C**) leaf MDA content 24 h after spraying with externally supported amino acids. CK, samples of exogenous water; Pro, samples of 10 mM exogenous proline; GABA, samples of 1 mM exogenous γ-aminobutyric acid. The data are the mean ± SD (*n* = 3). Significance was verified by *t*-tests; ‘*’ represents significance at *p* ≤ 0.05.

Under drought conditions, *CsProDH1* overexpression in tea plants for 24 h was followed by exogenous application of amino acids for recovery treatment. The effect of transient overexpression of the *CsProDH1* gene in Supplementary Materials (Figure S6). The results showed that groups treated with exogenous Pro and γ-aminobutyric acid experienced less stress compared to the control, as indicated by the fluorescence images (Figure 6A). The group treated with γ-aminobutyric acid had the fewest blue spots on leaves and the lowest MDA content (Figure 6B,C).

The effects of *CsProDH1* silencing on the content of different amino acids in tea leaves are shown in Table 1. Compared to normally grown tea plants, the silenced group exhibited significantly increased levels of serine, Glu, cysteine, leucine, tryptophan, histidine, arginine, and Pro. The effects of exogenous Pro application on the content of different amino acids in tea leaves are also shown in Table 1. After application of 10 mM exogenous Pro, compared to the 0 mM group, histidine was significantly reduced, while contents of methionine, tryptophan, and lysine significantly increased.

## 3. Discussion

Under abiotic stress, the dynamic changes in proline play a crucial role in plants. As a multifunctional amino acid, Pro not only acts as an osmotic regulator to help plants maintain cellular water balance but also functions as an antioxidant to scavenge ROS, thereby protecting cells from damage [37]. In *A. thaliana*, the increase in proline content during heat stress is associated with the upregulation of the gene for P5CS [38]. This accumulation helps protect cellular structures from heat-induced damage and maintains cellular redox balance. In peanuts, drought stress leads to proline accumulation, which, together with the increased activity of antioxidant enzymes, helps plants maintain cellular osmotic balance and antioxidant defense [39]. Additionally, plants such as wheat and tobacco increase Pro biosynthesis under drought stress by upregulating expression of Pro synthesis-related genes, such as those for P5CS and P5CR [40]. The accumulation of Pro helps plants maintain normal physiological functions in water-deficient environments, enhancing their drought resistance [41]. The tea plant, a globally significant cash crop, holds substantial economic and cultural importance [42]. According to the Food and Agriculture Organization of the United Nations, the global tea trade was valued at approximately USD 9.5 billion in 2022, with its distinctive flavor profiles and cultural symbolism captivating consumers worldwide. Despite their preference for humid environments throughout their life cycle, tea plants frequently encounter drought stress—a major agricultural constraint that compromises global tea quality and yield [43,44]. Drought exposure prolongs vegetative growth, reduces germination rates, and induces plant mortality. Empirical studies indicate yield reductions of 14–33% and mortality increases of 6–19% under drought conditions [45]. In our study, tea plants downregulated *CsProDH1* to reduce Pro degradation in response to drought stress.

Two relevant genes, *AtProDH1* and *AtProDH2*, have been identified in the *A. thaliana* genome [46]. These genes encode enzymes with different functions and regulatory mechanisms in Pro metabolism [47]. The *AtProDH1* is located in the mitochondria and participates in the oxidative metabolism of Pro. When Pro is in excess, AtProDH1 can oxidize proline to P5C, which is further converted to Glu, providing energy and nitrogen sources for the cell [48]. In this study, we found that the expression pattern of *CsProDH1* in tea plants is similar to that of *AtProDH1* in *A. thaliana* [21] (Figure 1B). Subcellular localization experiments revealed that the protein product of this gene was also located in the mitochondria (Figure 3). Based on their similarities, we speculate that *CsProDH1* in tea plants may have similar metabolic regulatory functions to *AtProDH1* in *A. thaliana*. In our study, the optimal pH range for *CsProDH1* in tea plants was 3.0–12.0, with the best pH value being 10 (Figure 2G,H). The optimal pH for *Arabidopsis* AtProDH was also reported to be 10 [49], confirming the phylogenetically conserved alkaline catalytic preference in the plant ProDH family. Enzymatic characteristic analysis showed that the Km value of CsProDH1 is 0.7180 mM, significantly lower than reported homologs from microbial and plant sources: *Pseudomonas fluorescens* PfProDH5 (35 mM), *E. coli* EcProDH (42 mM), *Thermus thermophilus* TtProDH (27 mM), and *A. thaliana* AtProDH (31 mM) [50,51,52,53,54]. This ultra-low Km value indicates evolutionary specialization in substrate binding affinity [55], likely through adaptive modifications in catalytic pocket architecture. Such kinetic superiority enables CsProDH1 to maintain catalytic efficiency under low Pro concentrations, ensuring rapid Pro degradation upon stress relief while fulfilling the physiological requirement for swift metabolic regulation during drought adaptation in tea plants. Furthermore, the optimal temperature for CsProDH1 enzymatic activity was 30 °C, while retaining partial functionality under 60 °C heat stress, demonstrating moderate thermotolerance (Figure 2E,F). Compared to the extremely thermophilic TtProDH from *Thermus thermophilus* (maintaining >85% and >60% residual activity after 1 h and 3 h at 90 °C, respectively) [50], CsProDH1 showed relatively lower thermal stability. Nevertheless, its heat-resistant properties may confer metabolic buffering capacity to tea plants under seasonal high-temperature conditions. Under drought stress conditions, the Pro content in tea plants significantly increased, and CsProDH1 could regulate the Pro metabolic process. Therefore, its enzymatic characteristics suggest that CsProDH1 may play an important role in regulating the drought resistance of tea plants.

Chlorophyll fluorescence measurements with OJIP analysis have been commonly used to study plant responses to abiotic stresses [56,57]. Our results showed that the K-phase and J-phase of the OJIP curve increased after overexpression of the *CsProDH1* gene compared to the control (Figure 4F); and the K-phase, J-phase, and I-phase of the OJIP curve decreased after silencing of the *CsProDH1* gene relative to the control (Figure 5F). In this study, to ensure the impact of different treatments on the K-band, the O-band (20 μs) and J-band (2 ms) were used to normalize the chlorophyll a fluorescence rise kinetics, and the W_OJ_ = (F_t_ − F_0_)/(F_J_ − F_0_) on a linear time scale from 0 to 2 ms. Previous studies demonstrated that drought stress induces the appearance of the K-band, and the K-band is an indicator parameter for OEC destruction [58,59,60]. Our study showed that under drought stress conditions, *CsProDH1* overexpression led to an increase in the K-band (Figure 4G and Figure 5G). The increase in the K-band indicates deactivation of the OEC center on the donor side of PSII [61]. This confirms that inhibiting *CsProDH1* expression under drought stress can lead to the accumulation of Pro and other amino acids within the cell, thereby preventing the reduction in PSII reaction center activity, reducing damage to the OEC center, enhancing the efficiency in converting light energy, and protecting PSII and PSI of the leaves, thus maintaining stability of the photosynthetic system. Chlorophyll fluorescence parameters and OJIP curves can be used as effective indicators for screening drought-tolerant genotypes of tea tree varieties and selecting and breeding drought-adapted varieties.

**Figure 7:**
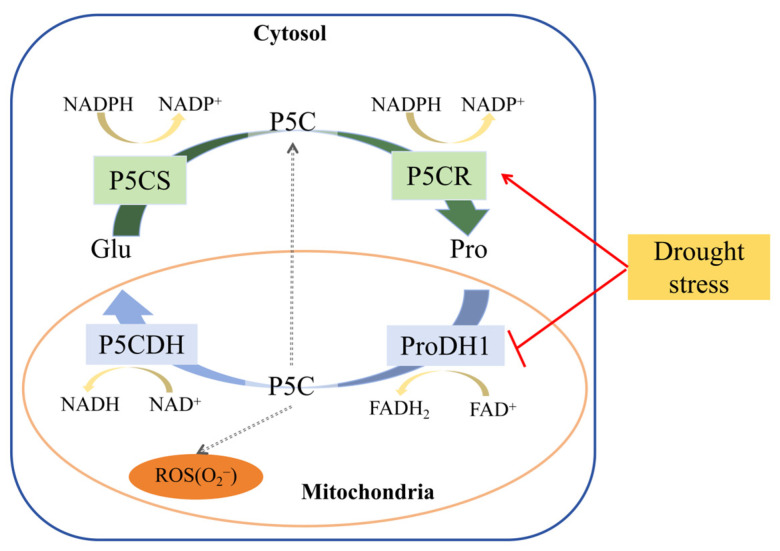
A model of ProDH-mediated regulation of Pro-P5C cycle homeostasis under drought stress. ProDH, proline dehydrogenase; P5CDH, Δ^1^-pyrroline-5-carboxylate dehydrogenase; P5CR: Δ^1^-pyrroline-5-carboxylate reductase; P5CS: Δ^1^-pyrroline-5-carboxylate synthase; Pro, proline; Glu, glutamate; P5C: Δ^1^-pyrroline-5-carboxylate; ROS, reactive oxygen species.

The Pro-P5C cycle primarily involves two key enzymes: ProDH and P5CDH. In the cycle, ProDH oxidizes Pro to P5C, transferring electrons to the mitochondrial electron transport chain, providing energy for the cell. Subsequently, P5C can be further oxidized to Glu by P5CDH or reduced back to Pro by P5CR in the cytoplasm, forming a cycle [62]. This cycle not only plays a role in energy metabolism but is also closely related to the redox balance within the cell. However, some studies have shown that excessive accumulation of Pro can increase ROS production through the Pro-P5C cycle, thereby having a negative impact on the cell. For example, accumulation of Pro under heat stress reduces thermotolerance, possibly by increasing ROS production through the Pro-P5C cycle [63]. Under stress conditions, wild-type plants exhibited stronger adaptability compared to transgenic *A. thaliana* plants [64], indicating that *CsProDH* overexpression may reduce the stress resistance of tea plants, which is detrimental to their growth. When ProDH activity increases disproportionately to P5CDH activity, the cycle operates, leading to incomplete oxidation of Pro and accumulation of P5C in the mitochondria [34]. Therefore, when *CsProDH1* was overexpressed, the oxidation of Pro and accumulation of P5C under drought stress in tea plants increased ROS production and caused cellular damage (Figure 4B and Figure 6B). In contrast, in transgenic tobacco, which has reduced NtProDH activity, it showed greater resistance to drought stress [65]. Our study confirms that proline overaccumulation may trigger ROS accumulation through an incomplete oxidative cycle, challenging the traditional simple model of more proline for better resistance and promoting finer metabolic homeostasis studies (Figure 7). Compared with model plants or crops, the study of the tea plant fills the gap of Pro metabolism in the field of economic forest trees [66,67,68,69], and the discovery of the tea plant not only expands the diversity of knowledge of plant stress adaptation but also provides a specific strategy for the breeding of agricultural stress tolerance.

*ProDH* expression is regulated by low osmolarity and proline signaling. Studies have shown that its transcriptional activation is mainly mediated by the basic leucine zipper (bZIP) family of transcription factors: members of the S1 subclass (bZIP2, bZIP11, bZIP44, and bZIP53) synergistically activate *ProDH* gene expression through the formation of a functional heterodimer with the C subclass factors (bZIP10 and bZIP6), which specifically recognizes the ACTCAT promoter motif and thus synergistically activates *ProDH* gene expression [70,71]. Notably, the cis-acting element PRE (proline-responsive element), which can be activated by intracellular proline accumulation or osmotic pressure reduction signals, significantly enhances the transcriptional activity of *ProDH* under rehydration conditions by interacting with ACTCAT motifs [72]. In addition to the above regulatory networks, *ProDH* expression is finely regulated by osmotic pressure fluctuations and hormonal signaling. For example, in *A. thaliana*, the response regulator ARR18 can repress *AtProDH1* transcription by competitively binding to specific regions of the promoter and destabilizing the bZIP53/bZIP63 heterodimer. This ARR18-mediated negative regulatory mechanism effectively reduces Pro catabolism and promotes intracellular accumulation under osmotic stress [73]. In the future, we can analyze the composition and distribution of cis-acting elements in the *CsProDH1* promoter region of tea plants by bioinformatics means to predict the reciprocal transcription factors; integrate the transcriptome data of tea plant stress; construct co-expression networks to screen the core regulatory factors of *CsProDH1*; and establish a gene editing and transgenic verification system to reveal the biological functions of key factors in the drought response of tea plants. These studies not only provide new perspectives for analyzing the molecular mechanism of stress tolerance in economic crops but also lay the theoretical foundation for the precision breeding of tea plants for stress tolerance.

## 4. Materials and Methods

### 4.1. Plant Materials

This study used one-year-old cuttings of the tea plant variety ‘Longjing 43’, purchased from Yarun Tea Industry Co., Ltd. in Nanjing, Jiangsu Province. Longjing 43 is a cultivated tea variety in China and is also commonly used in biological research. Tea seedlings with consistent growth were selected for hydroponic cultivation and subjected to different treatments [74]. They were placed under the following conditions: 16 h/8 h day/night photoperiod, light intensity of 30 klx, and 75~80% relative humidity. All tea seedlings were pretreated under the above conditions for 1 month. Subsequently, healthy tea seedlings without significant differences were selected as treatment materials.

The healthy tea seedlings pretreated for 1 month were transferred to either the full nutrient solution as a control group or the full nutrient solution with 20% (*w*/*v*) polyethylene glycol 6000 added as a drought group, as described previously. The following treatments were applied to both groups: 20 μM antisense oligonucleotide (asODN) was injected on the abaxial surface of leaves in the silent group, and *CsProDH1*-GFP suspension was injected on the abaxial surface of leaves in the overexpression group.

Healthy tea seedlings pretreated for 1 month were treated with exogenous amino acids as described previously. They were then sprayed with 10 mmol·L^−1^ of exogenous Pro [75,76], 10 mmol·L^−1^ of exogenous Glu [77], and 1 mmol·L^−1^ of exogenous γ-aminobutyric acid as experimental groups [78,79,80], while the control group was sprayed with an equal volume of water. Reference was made to the concentration of exogenous amino acids, and pre-experiments were carried out (Figures S1–S3). Each treatment group consisted of three pots, with four seedlings per pot. After 24 h of treatment, the top three leaves of the tea seedlings were collected, wrapped in aluminum foil, and quickly frozen in liquid nitrogen before being stored at −80 °C. Photos were also taken to document the phenotypic changes in tea plants before and after treatment.

### 4.2. Bioinformatics Analysis and qRT-PCR Analyses

The conserved sequences of the tea plant proline metabolism genes and their encoded amino acid sequences were analyzed as follows: Amino acid multiple sequence alignment analysis was performed using software such as Clustal X 2.1 and GeneDoc 2.7, and a phylogenetic tree was constructed using MEGA 6.0.

The expression of the tea plant *CsProDH* gene was analyzed by quantitative real-time PCR (qRT-PCR). Samples were selected from mature leaves (first, second, and third leaves). Total RNA was isolated from tea using the RNAprep Pure Plant kit (Bio TeKe, Wuxi, China) according to the manufacturer’s instructions. The RNA samples were reverse transcribed using the ReverAid First Strand cDNA Synthesis Kit (Thermo Scientific, Waltham, MA, USA). Quantitative data analysis was performed using the 2^−ΔΔCt^ method, with Csβ–actin serving as the reference gene. The Csβ–actin forward primer was GCCATATTTGATTGGAATGG, and the reverse primer was GGTGCCACAACCTTGATCTT [81,82]. The primer pairs used in this study are listed in Table S1.

### 4.3. Antisense Oligodeoxynucleotide Inhibition Assay

The sequence of tea plant proline dehydrogenase (*CsProDH1*, TEA027545) was input into the Soligo software “http://sfold.wadsworth.org/cgi-bin/index.pl (accessed on 28 March 2023)” to screen for asODN candidate sequences complementary to the target gene fragment, with ddH_2_O serving as the control. To design asODNs homologous to the target gene sequence, the most suitable asODN binding sites were selected based on nucleotide composition (%GC) and oligonucleotide binding energy (kcal/mol). To ensure and enhance the interference effect, three asODN sequences were designed and synthesized. The asODNs were synthesized by GenScript Biotech Co., Ltd. (Wuhan, China). The three synthesized asODN sequences were all dissolved in ddH_2_O and mixed to form the asODN injection solution. A volume of 500 μL of the asODN gene solution was injected into tea plant leaves to silence the gene in the leaves [83]. Each treatment and control group underwent three experimental replicates. The primer pairs used in this study are listed in Table S1.

### 4.4. Plasmid Construction of Overexpression Vector and Agrobacterium Infiltration

Based on the gene sequence of *CsProDH1* in the tea plant genome database, primers were designed using Primer Premier6.0 software for constructing the plant expression vector PBI121-GFP, which is preserved in our laboratory. The primers were designed to target the Xba I and BamH I restriction sites of the vector. All primers were synthesized by Nanjing GenScript Biotech Co., Ltd. in China.

Transformed Agrobacterium was grown in LB liquid medium using the above-obtained, and then the bacteria were resuspended in suspension (MES 25 mM, 2 mM Na_3_PO_4_, and As 150 µM), vortexed and shaken to mix well, and the solution was diluted to an OD600 of about 1.0 [84]. The suspension was aspirated using a 10 mL syringe and injected into the dorsal lower epidermis of well-grown tea seedling leaves and incubated normally for 1 day. Each treatment and control group underwent three experimental replicates.

### 4.5. Subcellular Localization Analysis

Predicting the location of CsProDH1 through multiple sites, including WoLF PSORT II, Cell-Ploc, and TargetP [85,86,87]. The *CsProDH1*-GFP fluorescent expression vector plasmid was transformed into Agrobacterium tumefaciens competent cells using the freeze–thaw method. After PCR verification of the correct transformation, subcellular localization was performed. The resulting *CsProDH1*-GFP single-colony *Agrobacterium* was cultured overnight at 28 °C in liquid LB medium, then resuspended in suspension solution (MES 10 mM, MgCl_2_ 10 mM, and As 150 µM) and mixed thoroughly. After a dark treatment of 3 to 5 h, the suspension was injected into the abaxial epidermis of tobacco leaves. The leaves were then cultured in the dark for approximately 16 to 20 h and normally cultured for 2 to 3 days. Fluorescence signals were observed and photographed using a laser confocal microscope. The primer pairs used in this study are listed in Table S1.

### 4.6. Protein Extraction, Electrophoresis, and Purification

Select the bacterial colonies containing the successfully constructed prokaryotic expression vector CsProDH1-GST and incubate them overnight at 37 °C. Inoculate the bacteria into LB liquid medium at a 1:100 volume ratio and shake until the optical density at A_600_ = 0.6. Then, add IPTG to a final concentration of 0.4 mmol·L^−1^ and induce at 37 °C for 0, 3, and 5 h. Collect a small amount of bacterial lysate for SDS-PAGE analysis. Select the bacterial colonies containing the successfully constructed prokaryotic expression vector CsProDH1-His and incubate them overnight at 37 °C. Inoculate the bacteria into LB liquid medium at a 1:100 volume ratio and shake until A_600_ reaches 0.6. Then, add IPTG to a final concentration of 0.4 mmol·L^−1^ and induce at 37 °C for 6 h. The purification steps for His-tagged proteins are described in the manual of the His-tag Protein Purification Kit (Beyotime, Shanghai, China). The primer pairs used in this study are listed in Table S1.

### 4.7. Enzymatic Characterization

The purified protein was transferred to a new tube for in vitro functional verification. For the control group (Mock), His-tagged empty protein was added; for the experimental group (ProDH1), CsProDH1 protein was added. The reaction mixture contained 1.6 mL of 0.15 mmol·L^−1^ Na_2_CO_3_–NaHCO_3_ (pH 10.3) buffer, 0.2 mL of 0.1 mol·L^−1^ L-proline solution, and 0.2 mL of 0.9 mmol·L^−1^ 2,6-dichlorophenolindophenol. After incubation at 30 °C for 5 min, 0.5 mL of enzyme extract (0.8 mg protein·mL^−1^) was added, and the mixture was thoroughly mixed. Then, 0.2 mL of freshly prepared 9 mg·mL^−1^ PMS was added, and the mixture was shaken and immediately measured for optical density changes at 600 nm using a spectrophotometer. After 30 min of reaction, the fully reacted solution was measured for P5C content using a microplate reader.

For enzyme activity determination, the reaction mixture contained 1.6 mL of 0.15 mmol·L^−1^ Na_2_CO_3_–NaHCO_3_ (pH 10.3) buffer, 0.2 mL of 0.1 mol·L^−1^ L-proline solution, and 0.2 mL of 0.9 mmol·L^−1^ 2,6-dichlorophenolindophenol. After incubation at 30 °C for 5 min, 0.5 mL of enzyme extract (0.8 mg protein·mL^−1^) was added, and the mixture was thoroughly mixed. Then, 0.2 mL of freshly prepared 9 mg·mL^−1^ PMS was added, and the mixture was shaken and immediately measured for optical density changes at 600 nm using a spectrophotometer. One unit of enzyme activity (1 U) was defined as 0.01ΔA_600_·g^−1^FW·min^−1^.

In the enzyme-catalyzed reaction solution, a series of proline concentrations ranging from 1 mM to 100 mM were used, while the concentrations of other reagents remained unchanged. Enzyme activity was measured as described in this section. The reaction velocity and substrate concentration were fitted with a linear equation, and a Lineweaver–Burk plot was constructed. The Vmax and Km of CsProDH1 were calculated based on this equation.

The temperature range of 20–50 °C was selected to measure enzyme activity as described in this section. The pH range of 3.0–12.0 was selected to measure enzyme activity as described in this section. The enzyme was incubated at temperatures ranging from 20 to 50 °C for 30 min, and then enzyme activity was measured as described in this section. The enzyme was incubated at pH values ranging from 3.0 to 12.0 at 4 °C for 12 h, and then enzyme activity was measured as described in this section.

### 4.8. Measurement of Physiological Indicators

Amino Acid Content Determination: Samples were ground into a powder using liquid nitrogen. A 0.2 g portion of the ground sample was placed in a centrifuge tube, and 2 mL of 0.02 mol·L^−1^ HCl was added. The mixture was shaken to ensure thorough mixing. After extraction at 4 °C for 8 h, the solution was centrifuged at 4 °C and 15,000 rpm for 15 min. Carefully, 2 mL of the supernatant was transferred to a new centrifuge tube, and an equal volume of 4% sulfosalicylic acid was added. The mixture was shaken again to ensure thorough mixing. Approximately 1.5 mL of the solution was then filtered through a 0.22 μm organic filter membrane into a small brown bottle and analyzed for amino acid composition using an amino acid analyzer (Hitachi L-8900, Tokyo, Japan).

P5C Content Determination: Approximately 0.2–0.3 g of leaf sample was homogenized in 10 mL of 3% sulfosalicylic acid. The supernatant was collected by centrifugation. Moreover, 1 mL of the supernatant was mixed with 0.1 mL of trichloroacetic acid, and the reaction mixture was prepared by adding 0.5 mL of 2-aminoacetophenone in 95% ethanol and incubating for 1 h. After preparation, the mixture was centrifuged at 12,000× *g* for 10 min, and the clear supernatant was collected. The absorbance was measured at 440 nm using a microplate reader [88].

The O_2_^−^ content was determined using a reagent kit (Solarbio, Beijing, China) according to the manufacturer’s instructions. The content of MDA was determined using a reagent kit (Solarbio, Beijing, China) according to the manufacturer’s instructions.

Staining of O_2_^−^ in tea tissue using the NBT method: Several tea leaves were cut using scissors and placed in a Petri dish. NBT reaction solution (2 mg·mL^−1^, pH = 7.8) was poured over the leaves, and the dish was wrapped with aluminum foil to protect it from light. The leaves were stained for 8 h. A decolorizing solution was then prepared with a ratio of ethanol: glacial acetic acid: glycerol = 3:1:1 (*V*/*V*). The leaves were completely submerged in this decolorizing solution and placed in a boiling water bath for 40 min until they lost all their green color. The leaves were then removed and immersed in 95% ethanol to wash away any remaining chlorophyll. The stained leaf tissues were observed and photographed.

### 4.9. Fast Chlorophyll Fluorescence-Induced Kinetic Curves and Fluorescence Images

The measurement site was the middle of the second leaf with healthy and uniform growth, and five plants were measured for each treatment. The leaves were dark-adapted for 30 min, and then the rapid chlorophyll fluorescence-induced kinetic curves (OJIP) were determined, and fluorescence images were taken with a Pocket PEA Plant Efficiency Analyzer (Hansatech, Norfolk, UK) and an IMAGING-PAM Chlorophyll Fluorometer (Walz, Effeltrich, Germany) [89,90].

### 4.10. Statistical Analysis

The significance of the data was statistically assessed by *t*-test with *p* ≤ 0.05 for repeated experiments. At least three biological replicates were determined for each experiment (*n* = 3). All data were statistically analyzed and tested for significance using Excel 2003 and SPSS 11.0, and GraphPad Prism 8 was used for graphing.

## 5. Conclusions

This study provides a comprehensive analysis of the function of the proline dehydrogenase gene *CsProDH1* in tea plants, particularly its importance in regulating the Pro-P5C cycle and coping with drought stress. Based on the findings of this study on the function of tea tree CsProDH1 in drought stress and its enzymatic properties, the molecular basis of its ultra-low Km value can be revealed in the future by resolving the three-dimensional crystal structure of CsProDH1, clarifying the amino acid composition of its substrate-binding domain, and exploring how plant ProDH has improved its substrate affinity through structural optimization in its evolution. Since systematic studies on the enzymatic properties of plant ProDH are still scarce (existing reports focus on model plants), we suggest cross-species comparative analyses as a direction for future research, especially for the adaptive evolution of ProDH in plants of different ecotypes. Explore how the activity balance between CsProDH1 and downstream enzymes affects the metabolic flow of the proline-P5C cycle to avoid ROS bursts caused by P5C accumulation. In the future, it can also be combined with metabolomics analysis to clarify the association between proline degradation and redox homeostasis under drought stress.

Research on the Pro-P5C cycle offers a new perspective for understanding the stress adaptation mechanisms of tea plants. In-depth study of the regulatory mechanisms of this cycle can provide a theoretical basis and potential gene targets for improving stress resistance of tea plants. Additionally, investigating the interactions between the Pro-P5C cycle and other metabolic pathways in tea plants can help reveal the complexity of plant metabolic networks. It is also possible to co-express *CsProDH1* with antioxidant enzyme genes or osmotic regulator genes to construct a synergistic anti-stress pathway, which enhances the tea tree’s adaptation to the compound stress (drought + high temperature). Future work could focus on molecular mechanism analysis, metabolic network regulation, genetic engineering application, and cross-species comparison to comprehensively explore the potential of *ProDH* in tea plants adversity biology and, at the same time, promote the innovative development of breeding technology.

## Figures and Tables

**Table 1:** Free amino acid content in tea leaves under different treatments.

	asODN		Exogenous Proline (Pro)	
	CK	asODN	0 mM	10 mM
Asp	1.00	1.26	1.00	1.25
Ser	**1.00**	**1.91**	1.00	0.90
Glu	**1.00**	**1.51**	1.00	1.16
Gly	1.00	1.54	1.00	0.90
Ala	1.00	1.55	1.00	1.05
Cys	**1.00**	**1.89**	1.00	1.01
Val	1.00	1.51	1.00	0.89
Met	1.00	1.94	**1.00**	**1.24**
Ile	1.00	2.25	1.00	0.98
Leu	**1.00**	**1.81**	1.00	1.03
Tyr	**1.00**	**3.15**	**1.00**	**1.73**
Phe	1.00	0.86	1.00	0.77
GABA	1.00	0.90	**1.00**	**0.77**
Lys	1.00	1.63	**1.00**	**1.43**
His	**1.00**	**3.80**	**1.00**	**0.76**
Arg	1.00	1.72	1.00	1.07
Pro	1.00	1.85	/	/

Table 1. Free amino acid content in tea leaves under different treatments. Red and blue coloring indicate higher and lower relative metabolite abundances. Bold text indicates significant differences between CK and asODN samples, as well as between 0 and 10 mM samples (*p* < 0.05). CK, samples without silencing the *CsProDH1* gene; asODN, samples of silencing the *CsProDH1* gene.

## Data Availability

All data are available in the manuscript or the Supplementary Materials.

## Supplementary Materials

The following supporting information can be downloaded at: https://www.mdpi.com/article/10.3390/ijms26073121/s1.
