# Supplementary material for: The Proline Dehydrogenase Gene CsProDH1 Regulates Homeostasis of the Pro-P5C Cycle Under Drought Stress in Tea Plants
Source: Int J Mol Sci. 2025 Mar 28;26(7):3121. doi: 10.3390/ijms26073121 (PMC11988676; doi:10.3390/ijms26073121)
Supplement: Supplementary file 1 [file ijms-26-03121-s001.zip › ijms-3489965-supplementary.pdf]

Supplementary Materials

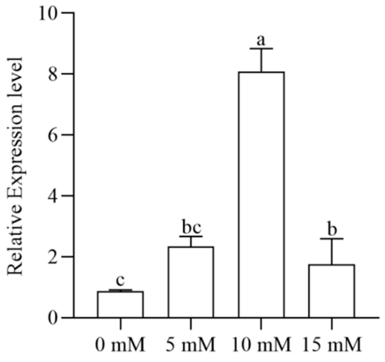

Figure S1. Validation of gene expression of *CsProDH1* after 24 h treatment with different concentrations of exogenous Pro. The data are the mean ± SD (n = 3). Significance was verified by one-way ANOVA, different small letters indicated there was a significant difference among different treatments ($P \leq 0.05$).

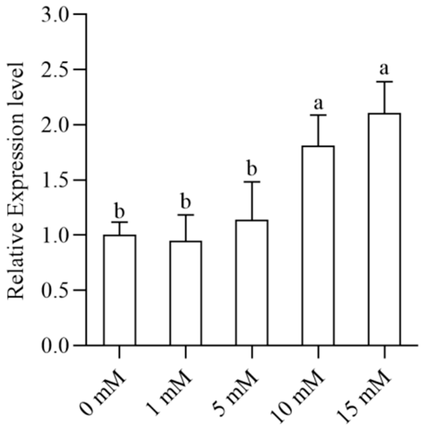

Figure S2. Validation of gene expression of *CsProDH1* after 24 h treatment with different concentrations of exogenous Glu. The data are the mean ± SD (n = 3). Significance was verified by one-way ANOVA, different small letters indicated there was a significant difference among different treatments ($P \leq 0.05$).

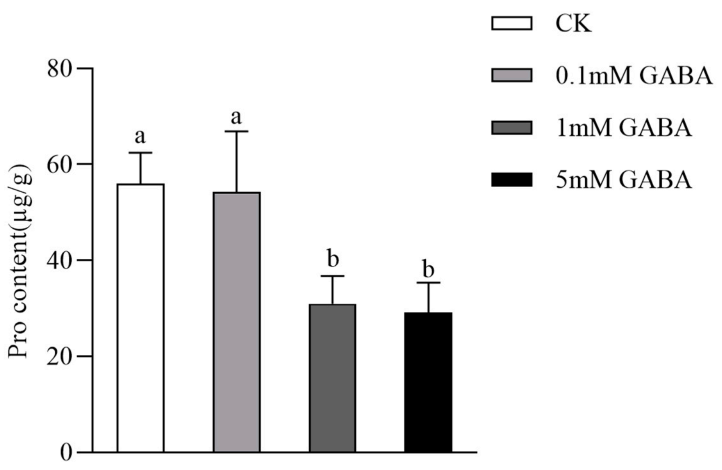

Figure S3. Pro content in tea plant leaves after 24 h of treatment with different concentrations of exogenous GABA under drought stress. CK, samples of exogenous water; 0.1 mM GABA, samples of 0.1 mM exogenous γ-aminobutyric acid; 1 mM GABA, samples of 1 mM exogenous γ-aminobutyric acid; 5 mM GABA, samples of 5 mM exogenous γ-aminobutyric acid. The data are the mean ± SD (n = 3). Significance was verified by one-way ANOVA, different small letters indicated there was a significant difference among different treatments ($P < 0.05$).

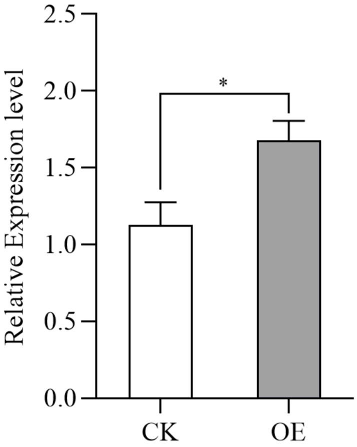

Figure S4. Validation of gene expression in transient overexpression treatment of *CsProDH1* in tea plant under drought conditions. The data are the mean ± SD (n = 3). Significance was verified by t-tests, '*' represents significance at $p \leq 0.05$.

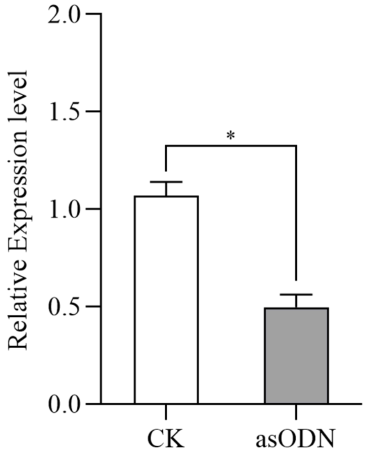

Figure S5. Validation of gene expression in transient silencing treatment of *CsProDH*1 in tea plant under drought conditions. The data are the mean ± SD (n = 3). Significance was verified by t-tests, '*' represents significance at $p \leq 0.05$.

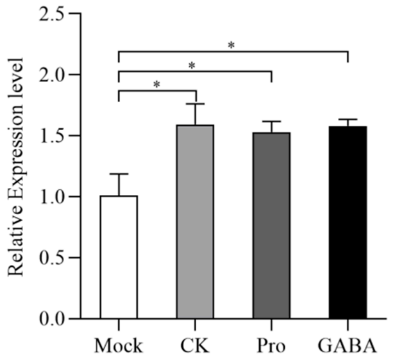

Figure S6. Gene expression validation of *CsProDH1* transient overexpression treatment before recovery treatment with exogenously applied amino acids. Mock, blank sample; CK, exogenous water sample; Pro, 10 mM exogenous proline sample; GABA, 1 mM exogenous γ-aminobutyric acid sample. The data are the mean ± SD (n = 3). Significance was verified by t-tests, '*' represents significance at $p \leq 0.05$.

Table S1. Primers used in this study.

Primer Name	Primer Sequence（5'-3'）	Remarks
qCsProDH1-F	ATGGCCAACCACCGTGTCTTC	qPCR
qCsProDH1-R	GAGAATTGCAGCCTTTAGT	qPCR
qCsProDH2-F	CTTCAATGATTGTGCCTCTT	qPCR
qCsProDH2-R	TCCTCTTCAACTCCTTCCT	qPCR
qCsP5CDH-F	AAGGCAGCTCACATGCTTTCCC	qPCR
qCsP5CDH-R	AGCCTGCTGGTAGCTCTTCGG	qPCR
qCsP5CR-F	GGAATGGGCCGGTCACAATCG	qPCR
qCsP5CR-R	CTTCCTCTGTTGCCGCTGCTC	qPCR
qCsP5CS-F	TAGCAGCGGCGGCGAGAC	qPCR
qCsP5CS-R	AGCCTAGACCCACCAGAACCAC	qPCR
qCsOAT-F	GGCTTGGCACGGACAGGAAG	qPCR
qCsOAT-R	AACCACTCCACCACGCAATGC	qPCR
Csβ-actin-F	GCCATCTTTGATTGGAATGG	Internal reference
Csβ-actin-R	GGTGCCACAACCTTGATCTT	Internal reference
CsProDH1-F	ATGGCCAACCACCGTGTCTTC	PCR
CsProDH1-R	GAGAATTGCAGCCTTTAGT	PCR
CsProDH1-X-F	ACACGGGGGACTCTAGAATGGCCAACCACCG TGTCTTC	PCR
CsProDH1-B-R	TGACCACCCGGGGATCCGAGAATTGCAGCCT TTAGT	PCR
CsProDH1-E-F	CGCGTGGATCCCCGGAATTCATGGCCAACCA CCGTGTCTTC	PCR

CsProDH1-E-R	GCTCGAGTCGACCCGGGAATTCGAGAATTGC AGCCTTTAGT	PCR
CsProDH1-E-F	GGCTGATATCGGATCCGAATTCATGGCCAACC ACCGTGTCTTC	PCR
CsProDH1-S-R	TGCGGCCGCAAGCTTGTCGACGAGAATTGCA GCCTTTAGT	PCR
AsODN-CsProDH1-1	GGTTTTCGCGGCGTGTGGTA	Gene suppression
AsODN-CsProDH1-2	TGCTAGGGGGCAAATTGCAG	Gene suppression
AsODN-CsProDH1-3	TCTTCTGGGGTTAGAGGGTT	Gene suppression
